# Case Report: Surgical Intervention for *Fasciolopsis buski* Infection: A Literature Review

**DOI:** 10.4269/ajtmh.20-0572

**Published:** 2020-09-21

**Authors:** Xinglang Wu, Weimin Wang, Qujin Li, Qiang Xue, Yue Li, Shengwei Li

**Affiliations:** Department of Hepatobiliary Surgery, The Second Affiliated Hospital of Chongqing Medical University, Chongqing, China

## Abstract

*Fasciolopsis buski*, also called the giant intestinal fluke, is the largest intestinal fluke of the zoonotic trematode parasites and found mainly in Southeast Asian countries, including China. *Fasciolopsis buski* infection was formerly a common health problem in many countries, but it is now rare. Typically, it can be cured by oral drugs, but some infected patients need surgical intervention because of the severity of their condition or because of an unclear diagnosis or even misdiagnosis. Here, we report a case of a 15-year-old girl from Guizhou Province, China, presenting with recurrent upper-middle abdominal pain that was misdiagnosed as a choledochal cyst. Through laparotomy combined with postoperative histopathological examination, the source of the pain was proven to be mechanical biliary obstruction caused by *F. buski* infection. In the past, mechanical obstruction, especially biliary obstruction, caused by *F. buski* infection leading to surgery was not uncommon, but it is very rare in modern society. Moreover, delayed treatment and misdiagnosis of parasitic infection can lead to severe consequences. Therefore, we reviewed the previous literature on *F. buski* infection treated by surgical operation and summarized the characteristics and therapeutic strategies of these cases to raise clinicians’ awareness of this rare infection.

## INTRODUCTION

*Fasciolopsis buski*, commonly known as the giant intestinal fluke, is a trematode of the Fasciolidae family and was first discovered in the duodenum of an Indian sailor by George Busk in 1843.^1^ It is now known to be a common intestinal parasite of humans and pigs in Asia, including southern and central China, and India.^2,3^ Infection with *F. buski* occurs through the consumption of raw or improperly cooked aquatic plants (water chestnut, water caltrop, water bamboo, etc.) contaminated with metacercariae, especially when peeling off the outer layers of the plant with human teeth.^4–6^ In addition, *F. buski* can also occur as a coinfection with other soil-transmitted helminths when the infected host is immunocompromised.^7^ Mild infection of *F. buski* is asymptomatic, but in heavy infection, the common symptoms are abdominal pain, diarrhea, low-grade fever, toxemia, allergic symptoms, anemia, ascites, generalized edema, and intestinal obstruction, sometimes even leading to death.^8,9^ In general, the diagnosis of *F*. *buski* is based on the detection of eggs in stool, but a definitive diagnosis requires the observation of adult worms.^10^ Oral praziquantel is effective in the vast majority of diagnosed patients, but some patients cannot be diagnosed or have severe symptoms and may require surgical treatment first.

In clinical practice, a rare kind of mechanical biliary obstruction caused by *F*. *buski* infection could sometimes be misdiagnosed and treated as other biliary diseases. Here, we report a 15-year-old girl presenting with recurrent upper-middle abdominal pain for 10 years. In addition, ultrasonography (USG) and magnetic resonance cholangiopancreatography (MRCP) suggested dilatation of the common bile duct. Finally, her illness was confirmed through exploratory laparotomy due to *F. buski* infection. To increase clinicians’ awareness about these kinds of cases, we reviewed the previous literature on *F. buski* infection treated by surgery (Table 1). Moreover, the characteristics and therapeutic strategies for *F*. *buski* infection are also summarized in the present study.

**Table 1:** Important characteristics of patients with *F. buski* infection treated by surgery

First author	Year	Country	Gender	Age (years)	Epidemic area	History of raw aquatic plants	Presurgery stool examination	Endoscopic examination	Imaging examination	*F. buski* diagnosed	Eosinophil increase	Symptoms	Worm location	Coinfection	Surgical forms	Worm load	Post-surgery stool examination	Post-surgery treatment	Outcome
Jin and Si^40^	1972	China	F	46	Yes	Yes	N/A	No	Venous cholangiography	No	N/A	Abdominal pain and distension, chills, fever	Duodenum and the CBD	*Ascaris lumbricoides*	Laparotomy	3	Ova (−)	Areca decoction	Recovered
	1973	China	M	66	Yes	Yes	Ova (+)	No	N/A	Yes	N/A	Upper abdominal pain and jaundice	CBD	*Ascaris lumbricoides*	Emergency laparotomy and decompression of the biliary tract	2	N/A	Okra fried pepper and areca decoction	Recovered
	1974	China	M	41	Yes	Yes	Ova (+)	No	N/A	Yes	N/A	Upper-middle abdominal colic, fever, chill, and jaundice	CBD	*Ascaris lumbricoides*, hookworm	Emergency laparotomy and decompression of the biliary tract	8	N/A	Pepper, areca decoction, and buphenine	Recovered
Zhou^33^	2002	China	F	24	Yes	Yes	Undone	No	USG	No	N/A	Right upper quadrant dull pain, abdominal distension, and fever	CBD	No	Open cholecystectomy and common bile duct exploration	3	Ova (+)	Oral praziquantel	Recovered
Wang et al.^41^	2004	China	F	46	Yes	Yes	N/A	No	X-ray	No	N/A	Constipation, abdominal pain, and distension	Jejunum and ileum	No	Emergency laparotomy	153	N/A	N/A	Recovered
Bhattacharjee et al.^42^	2009	India	M	10	Yes	N/A	N/A	No	X-ray	No	Yes	Central abdominal pain and abdominal distension fever	Ileum	No	Emergency laparotomy and ileostomy	More than 25	Ova (+)	Oral praziquantel	N/A
Singh et al.^30^	2011	India	F	22	Yes	N/A	N/A	No	X-ray	No	Yes	Constipation, abdominal distension, fever, and vomiting	Jejunum and ileum	No	Emergency laparotomy, ileostomy, and end-to-end anastomosis	Heavy loads	Ova (−)	Oral praziquantel	Recovered
Ma and Yang^43^	2017	China	F	32	Yes	Yes	N/A	No	Magnetic resonance imaging	No	Yes	Right upper quadrant pain	Left hepatic duct and the CBD	No	Laparotomy and biliary tract exploration	2	Ova (−)	Oral praziquantel	Recovered
Wu et al.	2020	China	F	15	Yes	Yes	Ova (−)	Yes	USG and magnetic resonance cholangiopancreatography	No	Yes	Recurrent upper-middle abdominal pain	CBD	No	Laparotomy and biliary tract exploration	1	Ova (−)	Oral praziquantel	Recovered

CBD = common bile duct; F = female; *F. buski* = *Fasciolopsis buski*; M = male; N/A = not applicable; USG = ultrasonography.

## CASE PRESENTATION

**Figure 1.**
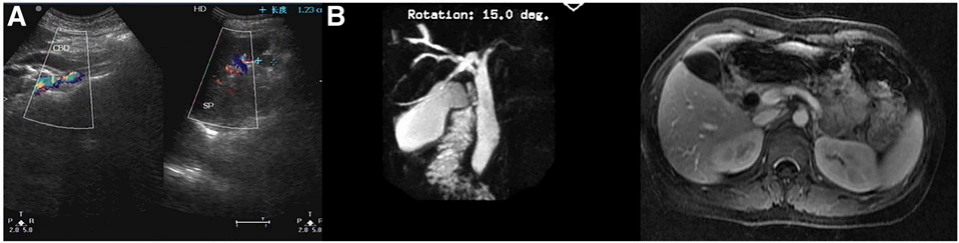
(**A** and **B**) Ultrasonography and magnetic resonance cholangiopancreatography showing dilatation of the common bile duct. This figure appears in color at www.ajtmh.org.

**Figure 2.**
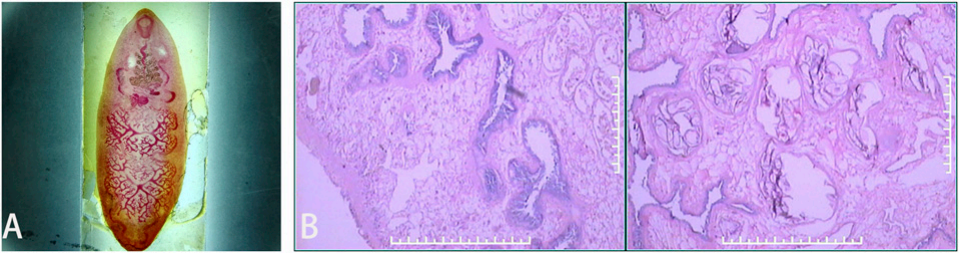
(**A**) *Fasciolopsis buski* worm, measuring 1.6 × 1.0 × 0.1 cm, in the common bile duct. (**B**) Histopathological and sectional photograph of *F. buski*. This figure appears in color at www.ajtmh.org.

A 15-year-old Han Chinese girl from Guizhou Province was admitted with a history of recurrent upper-middle abdominal pain for 10 years that had intensified for 4 days. The abdominal pain started 10 years previously with no obvious predisposing causes, lasted for approximately a few minutes, and went away on its own. Her parents took her to the local hospital several times, and she was suspected of having cholangitis due to intrahepatic stones. However, the condition did not improve during the course of the treatment. After admission, the doctor took a careful history, and the girl denied a history of parasites, viral hepatitis, travel, and direct contact with live animals or poultry. On physical examination, she was mildly anemic and malnourished, and the abdominal examination showed light tenderness in the upper-middle abdominal region. Routine blood examination revealed a hemoglobin level of 116 g/L, neutrophils 71.1%, and eosinophils 8.0%. In addition, liver function tests showed an alkaline phosphatase (ALP) level of 152 U/L, and bilirubin was normal. Routine stool and occult blood tests showed no abnormal changes, including parasitic eggs. Ultrasonography and MRCP showed dilatation of the common bile duct and a right intrahepatic stone (Figure 1A and B). The gastroduodenoscopy only revealed superficial gastrosinusitis. Combined with the aforementioned clinical symptoms and examination results, choledochal cysts and right intrahepatic stones with cholangitis were suspected. Symptomatic therapy was administered by using antibiotics, analgesic drugs, and stomach- and liver-protective drugs. The patient achieved remission after treatment but still felt unbearable irregular pain, and the diagnosis was still unclear. Because of economic and personal reasons, her parents decided on exploratory laparotomy rather than laparoscopy. Except for slight edema of the gallbladder and a slight thickening of the common bile duct and its wall, no tumor or other abnormalities were found in the abdominal cavity. Therefore, only common bile duct exploration was performed (the gallbladder was preserved). After opening the common bile duct, surprisingly, the stone forceps removed a flat foreign body from the middle of the duct, measuring 1.6 × 1.0 × 0.1 cm, with a sucker at the front and a tail-like object at the back (Figure 2A). Careful observation showed that the object could move and was considered as a biliary parasite. Choledochoscopic examination revealed that the confluence of the hepatic duct and the lower segment of the common bile duct were covered with green pus. In addition, no other worms were in the biliary tree, and the Oddi sphincter was in good condition. A 20f T-tube drain was placed into the bile duct, and an abdominal drain was placed in the omental foramen. After the operation, there was mild abnormal liver function (ALP of 124 U/L and alanine aminotransferase of 43 U/L) and leukocyte increase (15.65 × 10^9^/L), which returned to normal after treatment with antibiotics and liver-protectant drugs. Postoperative histopathological examination of the specimen showed the presence of *F. buski* (Figure 2B), but no eggs were found in the stool after surgery. Without any severe postoperative complications, the abdominal drain was removed on the third postoperative day, oral praziquantel (15 mg/kg) was given in divided doses for 1 day on the sixth postoperative day, and the patient was discharged on the eighth postoperative day with a T-tube drain. Four weeks later, she returned to the hospital and had a normal T-tube removal after cholangiography. Regular follow-up showed that her stool was negative for ova of *F. buski*, and she remained asymptomatic.

## DISCUSSION

Fasciolopsiasis is a foodborne, intestinal, zoonotic, and anthroponotic snail-transmitted parasitosis caused by a trematode termed *F. buski*, the largest fluke parasitizing humans.^11^ It infects humans, pigs, dogs, and rabbits in Bangladesh, China, Taiwan, India, Indonesia, Malaysia, Thailand, the Lao People’s Democratic Republic, and Vietnam.^12,13^ *Fasciolopsis buski* remains a public health problem in endemic regions.^14^ Adult worms typically measure 2–10/0.8–3 cm in length and eggs measure 130–140/80–85 mm.^15^ The worms inhabit the duodenum and jejunum, and can also be found in much of the intestinal tract, including the stomach, in moderate and heavy infections.^16^ Then, immature eggs are released in the stool and embryonate in water in 3–7 weeks.^17,18^ After hatching, miracidia infect pulmonate planorbid snails of the genera *Segmentina* (*Trochorbis*), *Hippeutis* (*Helicorbis*), and *Gyraulus*, which after 4–6 weeks shed cercariae (which undergo several stages: sporocysts, rediae, and cercariae).^19^ Cercariae encysts are found not only on the surface of aquatic plants and debris but also on the water surface.^18^ Humans are infected by ingesting metacercariae on aquatic plants that develop into adult flukes in the intestinal tract of the mammalian hosts (humans and pigs) in approximately 3 months. If the stool of the infected host enters fresh water, the eggs hatch, releasing immature larvae (miracidia), which enter snails to begin the cycle again.^20,21^

In the last century, fasciolopsiasis was most prevalent in school-age children, and the prevalence of infection in children ranges from 57% in China^18,22^ to 25% in Taiwan, China,^23^ and from 60% in India^24^ and 50% in Bangladesh^25^ to 10% in Thailand.^16,26^ Moreover, the first national survey reported 2,353 cases across China, with a national prevalence of 0.2%.^27^ It was extrapolated that there were 1.91 million infections with this parasite in 17 provinces.^21^ *Fasciolopsis buski* prevalence did not differ between genders but was related to profession; farmers were at the highest risk of infection.^27^ According to the results of the second national survey, *F. buski* had considerably lost prominence as a human parasite.^28^ However, pockets of high endemicity still persist in some countries. In fact, the morbidity of fasciolopsiasis is associated with economic status, educational background, standard of health, and way of life.^29^ The risk factors may include practice of open defecation in field or river banks, and alongside ponds; eating of raw aquatic crops and infected snails; and washing of the kitchen utensils and raw vegetables in pond water.^16^ Therefore, poor health conditions and consumption of raw aquatic plants lead to high risk in epidemic areas. To date, with the improvement of economic conditions and changes in eating habits, *F. buski* infection has almost disappeared in most countries.

The clinical manifestation depends on the parasitic load in the gut, local pathological changes, and absorption of toxic allergic metabolites.^30^ Pathological changes may be traumatic, obstructive, and toxic, especially in heavy infections, in which worms disturb the secretion of intestinal juices, cause excess mucus secretion, and obstruct the passage of food.^13^ Most patients are asymptomatic or may present with various symptoms, such as abdominal pain, abdominal distention, diarrhea, poor appetite, vomiting, and anemia with eosinophilia.^17,30^ Heavy infection can be fatal and is associated with malnutrition, intestinal inflammation, intestinal perforation, small bowel stricture, ulceration, hemorrhage, and abscess formation.^20^ In addition, generalized toxic and allergic symptoms, usually in the form of edema, particularly of the face, abdominal wall, and lower extremities, can occur.^13,31^ With the case reports increasing, some symptoms that are not typical of the aforementioned have also appeared. If the adult worms move to the ileocecal region, shifting pain in the right lower quadrant can occur and can be misdiagnosed as appendicitis.^32^ If the infection moves to the biliary tract, it can cause cholangitis, jaundice, biliary dilatation, or obstruction, as seen in our case and Zhou’s case.^33^ In fact, *F. buski*, as an intestinal parasite, rarely migrates to the biliary tract, but *Fasciola hepatica*, which belongs to the same family, Fasciolidae, is indeed a biliary parasite, often leading to biliary obstruction, cholangitis, gallstones, sclerosing cholangitis, and so on.^34^ Therefore, we should focus on distinguishing it from other biliary parasites and biliary diseases.

In general, the diagnosis of *F. buski* is based on the detection of eggs in stool, but the differentiation between *F*. *buski* and *F*. *hepatica* is very difficult in routine examination of stool, so a definitive diagnosis requires the examination of adult worms.^8,10^ In addition, serological testing has been developed as an alternative for fecal examination; ELISA with an antigen of adult *F. buski* to detect specific antibodies in human sera showed high sensitivity and specificity and a low rate of cross-reactions with *Schistosomes* and *Paragonimus* spp.^21,35^ In addition, gastroscopy was proposed to diagnose and treat early-stage fasciolopsiasis infections. As the early clinical symptoms of *F. buski* lack specificity, gastroscopy can detect the infection in the gastrointestinal tract directly, even removing the worms under the guidance of an endoscope and completing the treatment of the infection.^21,36^ In terms of treatment, many drugs have been used. Previously, tetrachloroethylene, areca decoction, and niclosamide were effective. Later, praziquantel at a dose of 15 mg/kg alone showed efficacy even in severe fasciolopsiasis and became the first-line choice.^37–39^

In fact, the symptoms of most patients are obvious and can be clearly diagnosed, and after oral praziquantel treatment, they can be completely cured. However, some patients may not have a clear diagnosis or may even be misdiagnosed because of a small worm load or inconspicuous clinical symptoms, leading to unnecessary invasive surgery. Although there are few patients with very large worm loads or special parasitic positions, these types of infections may cause emergencies and even be life-threatening, requiring surgical intervention first. A large number of reports have involved conventional infection of *F. buski*, but there are few reports associated with these situations, such as difficult diagnoses, misdiagnoses, and surgical treatments. The characteristics of surgical intervention cases have not been summarized, so we performed a literature review combined with our case report.

We searched the PubMed database and China Knowledge Network data platform (http://www.Cnki.net) from inception through April 2020, using the following key words: “*F. buski*,” “operation,” and “misdiagnosis.” After exclusion, only six articles (eight cases) met our standards.^30,33,40–43^ Their clinical data and ours are summarized in Table 1. Among these patients, five (62.5%) were female, three (37.5%) male, and one pediatric case, and the age range was 10–66 years and the mean age was 35.88 years. All patients were from epidemic areas (China and India), and six patients had the habit of eating raw aquatic plants (there were no data on eating habits for two patients). Three patients had other parasitic infections, including *Ascaris lumbricoides* or hookworm. Seven patients (87.5%) presented with abdominal pain to different degrees, and abdominal distension, chills, fever, jaundice, constipation, and vomiting were also common. In terms of diagnosis, gastroscopy was not performed in any case except ours, and routine stool examination played an important guiding role in preoperative examinations. Although preoperative routine stool examination results (presence of eggs) were not mentioned in most of the literature, the results were reported after operation. Of course, the eosinophil results were high in all cases. Only organ damage occurred, and magnetic resonance imaging, ultrasound, and X-ray reflected the positive results. Five of the cases involved emergency operations, and it was not clear that the surgery was performed directly because of *F. buski* infection, even if preoperative misdiagnosis exists. According to the intraoperative exploration, there were five cases of adult worms attached in the biliary tract and three cases in the intestinal tract. The number of worms was at least two, with a maximum of 155. The operations were successful, except for in one case due to postoperative complications. In addition, all patients were given postoperative anthelmintic treatment, although the oral drugs used were different, and all patients recovered in the end. Indeed, we also found that several cases used endoscopy as an important diagnostic method and to extract adult worms from the gastrointestinal tract.^20,31,44^ In Sen Sarma’s case, *F. buski* presented as acute upper gastrointestinal bleeding in an 8-year-old girl from rural North India, and emergency duodenoscopy revealed a live 4-cm long, flesh-colored, tongue-shaped organism in the second part of the duodenum that was gently removed with endoscopic grasping forceps.^31^

In our case, we were not aware of the parasitic infection until the operation was performed because the girl had negative results of hepatitis, stool routine examination, and endoscopy. The patient was young and had recurrent abdominal pain for 10 years. Both USG and MRCP showed dilatation of the common bile duct, so the cause was considered to be a choledochal cyst, although this diagnosis was not consistent with her clinical manifestations. Finally, to demonstrate the etiology of the disease and to relieve symptoms, common bile duct exploration was performed, and *F. buski* adult worms were identified as the obstruction in the biliary tract. Then, we reviewed the disease history in detail again and found that the patient’s hometown was rich in water chestnuts and that she had the habit of eating them raw. In addition, Guizhou Province is an epidemic area of *F. buski*, although the morbidity is currently very low. Moreover, preoperative blood tests also indicated an increase in eosinophils. The negative results of routine stool examination (no eggs) and gastroscopy deceived us and brought about great difficulties in making the correct diagnosis. In modern society, biliary parasitic disease is very rare, and routine blood tests showed an increase in eosinophils, but due to the doctor’s lack of experience in this area, it did not attract enough attention.

This is a very interesting case, and we hypothesize that the worms may initially stay in the gastrointestinal tract and then migrate to the biliary tract and cause recurrent abdominal pain for many years. After the biliary tract was unobstructed and the patient was given anthelmintic treatment, she had no abdominal pain, her eosinophils returned to normal, and the routine stool examination was negative. Overall, based on the previous literature and this case, we can draw the following conclusions: 1) only a few *F. buski* infections can lead to biliary obstruction, misdiagnosis, severe organ damage, and even death; 2) when the gastrointestinal tract and biliary tract are mechanically obstructed by the adult worms, which endanger the patient’s life or seriously influence the quality of life, surgical treatment is required; and 3) priority should be given to endoscopy, which can be used for both diagnosis and treatment. Laparoscopy or laparotomy should be performed if necessary. 4) Anthelmintic treatment is still the key to treating *F. buski* infections, and avoiding eating raw water-derived food and improving health awareness are important prevention and control strategies. Although parasitic infections have become increasingly rare now, they cannot be ignored in endemic areas, such as hydatid disease in Sichuan Province.

## CONCLUSION

*Fasciolopsis buski* infections, especially infections with heavy worm loads or in particular locations (biliary tract, ileocecal, etc.), have become rare. Through the patient’s history, such as eating aquatic plants without boiling or living in an endemic area, an increase in eosinophils, and eggs in stools, the diagnosis can be determined easily. Only a few patients lack the aforementioned specific manifestations and are extremely difficult to diagnose. Furthermore, sufficient doses of oral praziquantel are effective for patients. However, in regard to situations that endanger the patient’s life or seriously affect the quality of life, early surgical intervention is an important therapy. It can even be preferred as the first choice in some cases, such as when the diagnostic evidence is insufficient, similar to our case. In the era of minimally invasive surgery, endoscopy or laparoscopy should be widely used and performed if necessary.
